# Purperal Fever

**Published:** 1830-07-01

**Authors:** 


### XLVII.

### MATERNITE.

### PUERPERAL FEVER.

M. TONNELÉ has published a long article on the above disease in the Archives for March and April, 1830, as observed in the MATERNITÉ Hospital, in the year 1829. Of many cases whose dissections are given, we shall notice the only four in detail, as bearing on a point of pathology now under investigation in this country.

*Case* 1. Mary B. aged 23 years, experienced on the second day after her accouchement, acute pains in the hypogastrium, with fever. Forty leeches were applied, and produced much relief. But the next day brought a renewal of the pains, to which were added abundant diarrhœa and frequent vomiting. The lochia became suppressed, the breasts collapsed. Sixty leeches were applied to the abdomen, and forty more in the course of the day. Next morning the patient appeared easy, and the lochia reappeared. On the 4th day there was great agitation and tendency to faintings. On the 5th delirium came on, and she complained of pain in the thigh, which was somewhat *œdematous* on the fore-part. On the 6th day, she began to expectorate blood and fetid matters—had involuntary motions—and rapidly sank.

On examination there was found much sero-purulent effusion, with false membranes in the cavity of the peritoneum. The cervix uteri and broad ligaments were infiltrated with pus. The majority of the uterine veins were gorged with the same fluid. The extremities of the fallopian tubes were injected and thickened— the ovaries enlarged and softened. There was a gangrenous focus in the right lung. To the above morbid appearances was added *suppuration of the whole leg affected*;—even the muscles were infiltrated with pus. No mention is made of any affection of the veins of the limb.

*Case* 2. G. ———, aged 37 years, of good constitution, experienced, on the 3d day after delivery, the symptoms of intense puerperal fever, which were met by most active treatment, and abated towards the eighth day; but the fever and the abdominal pain soon reappeared—great restlessness and delirium supervened—and the patient sunk on the fifth day of the relapse.

On dissection there were evidences of very intense uterine phlebitis, to which were added collections of purulent matter in the psoas muscles, which were softened in substance. There were no other alterations except some purulent depositions in the iliacus internus, and also in the triceps femoris.

*Case* 3. Eliz. Hain, aged 23 years, was seized with puerperal fever on the fourth day after her accouchement, and numerous leeches were applied which seemed to conquer the malady quickly; but on the fourteenth day after delivery, when she was in full convalescence, an intense pain took place in the hypogastric region, without any ostensible cause. Next day there was violent fever, vomiting, deep-seated pain in the hypogastrium, and in the left iliac fossa. Forty leeches were applied, and produced a temporary relief. Then succeeded a series of seven symptoms, which left little doubt as to the nature of the malady. Delirium, agitation, small quick pulse, fetid diarrhœa, deep-seated pain in the hypogastrium—and ultimately in the thighs and one arm, but without any swelling, redness, or other external phenomenon.

On dissection, the deep-seated muscles of both legs were infiltrated with thick pus, the muscular fibres in contact with the pus were softened, and there were several isolated purulent depôts, the size of an almond, in the substance of the muscles of the leg, especially in the tibialis anticus. There was a purulent depôt about the middle of one thigh. The right fore-arm presented similar appearances. There was some pus in the cavity of the left knee. The veins of the uterus were filled with pus, and their internal surface discoloured and rough. The left ovarium was transformed into a purulent depôt—adhered to the rectum, and opened into that gut by a small orifice. The peritoneum was natural, and all other parts unaffected.

*Case* 4. B.———, aged 23 years, of vigorous constitution, experienced, soon after delivery, the symptoms of uterine phlebitis, soon followed by those grave phenomena which result from pus being carried into the torrent of the circulation. Œdematous swellings in different parts of the limbs appeared, and the patient died.

On dissection, numerous purulent depôts were found in the muscles of both legs, thighs, and arms. The cavity of the left knee also contained much pus of a good quality, without any appreciable affection of the synovial membrane. Most of the uterine veins were filled with purulent matter, and the parietes of the womb were thickened and unequal. Several of the larger lymphatics were gorged with the same fluid.

The author, (who, no doubt, speaks the sentiments of M. Desormeaux) conceives that the phenomena above-mentioned, namely, the purulent depôts in various and distant parts of the body, cannot be attributed to common inflammation.

"But if, says he, there exists in the constitution a general cause, manifest, and, in some measure palpable, capable of explaining these various effects—if we can detect pus ready formed in the uterine vessels, whence we see it carried by the blood to all parts of the body—if characteristic symptoms announce, in an almost certain manner, this importation of pus into the bosom of all the organs, and constantly precede the abscesses in question, have we not a thousand reasons for believing that these purulent depôts in the lungs, liver, brain, &c. are not the effects of common inflammation, but the result of a peculiar operation, or simple deposition of matter in the midst of the muscular tissues."

The author anticipates an objection to the muscles of the extremities being affected in preference to those viscera where the venous circulation flows in great abundance, as the lungs, the liver, &c. but he appeals to certain experiments of M. Cruveilheir, who injected mercury into the veins of animals, and often found the foreign matter deposited in the middle of the muscles. He acknowledges that it is very difficult to account for this caprice, as it were, of the disease, in respect to the direction which it takes to one or other organ. But passing over many cases and arguments, we come to an important table exhibiting the results of 390 post-mortem examinations of puerperal fever, with the various appearances on dissection.

Of these 222 cases, there were 193 cases of peritonitis—197 do. exhibiting disease of the uterus and appendages.*

The alterations of the uterus and of the peritoneum were variously combined in 165 cases. They were isolated in 57 instances, viz:—there was peritonitis alone in 28 cases, uterine affection alone in 29 cases. In respect to alterations in the uterus, there were 79 cases of simple metritis—29 of superficial mollescence—20 of deep-seated mollescence—58 of ovarian inflammation—four of ovarian abscess. The following is a tabular and comparative view of alterations in the vessels. In 90 cases, there was pus in the veins—in 32, pus in the lymphatics—in five cases, pus in the thoracic duct—in nine instances there was inflammation and suppuration in the lumbar and other lymphatic glands.

SUPPURATION of the VEINS was accompanied by that of the uterus in 32 cases—by mollescence or putrescency of the uterus in 11 cases—by metritis and mollescence united, in five cases—by peritonitis purely, in 34 cases—and it (suppuration of the veins) was isolated, or unaccompanied by any other affection, in eight cases.

SUPPURATION of the LYMPHATIC VESSELS existed in conjunction with that of the veins, in 20 cases—with that of the uterus, in 13 cases—with mollescence alone of the womb, in six cases—with simple peritonitis, in three cases—without any other lesion, in two cases.

INFLAMMATION of the OVARIES was accompanied by simple peritonitis in 29 cases—with various lesions of the uterus, in 27 cases—with simple metritis, in eight cases—with mollescence of the uterus in seven cases—with suppuration of the vessels, in 12 cases—and with all of the above alterations combined, in 16 cases.

From the above tabular facts, it results that, in fatal puerperal fever, the uterus is rather more frequently affected than the peritoneum—that the two affections are, in a majority of cases, combined—but they are sometimes entirely isolated. The table also presents this remarkable fact that, in 134 cases, the venous or lymphatic vessels of the uterus contained pus. Whether this pus was formed in the vessels themselves, or carried thither by absorption from other parts, is a secondary consideration—for the danger is the same, in both cases, of a general contamination of the circulating fluid. That the pus is sometimes, at least, formed in the vessels themselves, appears to be proved by the fact that, in eight cases where pus was found in the veins, there was no other lesion whatever. This fact, the author thinks, may throw a doubt on the opinion that the pus is ever absorbed into the veins from other parts. The table in question may help to explain the great danger or mortality in puerperal fever—it may also tend to throw some doubt on the propriety of the term puerperal peritonitis, or puerperal metritis, which has been so often applied to the disease. The designation "PUERPERAL FEVER" is freer from objection than either of the above terms. It prejudges nothing.

* The reason why the aggregate of the two classes of affections exceeds in number, the total of dissection, is, that, in many cases there was a combination of peritonitis and metritis.—*Ed.*

